# Pharmacists’ perspectives on traditional, complementary, and integrative medicine in Japan with special reference to Kampo medicines: an internet survey with preliminary interviews

**DOI:** 10.1186/s40780-022-00238-x

**Published:** 2022-03-01

**Authors:** Yoshiharu Motoo, Keiko Yukawa, Kazuho Hisamura, Ichiro Arai

**Affiliations:** 1 Department of Medical Oncology and Kampo Medicine, Komatsu Sophia Hospital, 478 Oki-machi, Komatsu, Ishikawa 923-0861 Japan; 2 Department of Health Policy and Technology Assessment, National Institute of Public Health, 2-3-6 Minami, Wako, Saitama, 351-0197 Japan; 3 Department of Medical Oncology, Kanazawa Medical University, 1-1 Daigaku, Uchinada, Ishikawa 920-0293 Japan; 4 Department of Pharmaceutical Sciences, Nihon Pharmaceutical University, 10281 Ina, Komuro, Kita-Adachigun, Saitama, 362-0806 Japan

**Keywords:** Traditional, complementary, and integrative medicine (TCIM), Usability, Evidence-based Japanese integrative medicine (eJIM), Pharmacist, Japan, Kampo, Dietary supplement

## Abstract

**Background:**

Pharmacists guide patients in their use of traditional, complementary, and integrative medicine (TCIM). The present study aimed to evaluate the opinions of Japanese pharmacists regarding TCIM, and to evaluate the usability of the evidence-based Japanese Integrative Medicine (eJIM) website from the pharmacists’ point of view.

**Methods:**

We conducted a two-stage, mixed-method study using interviews and an internet-based survey. In-person interviews were conducted with 20 pharmacists working in hospitals, dispensing pharmacies, or retail pharmacies. We analyzed their perspectives on TCIM and the usability of the eJIM. Based on the interviews, questionnaires for an internet survey conducted in February 2019 were developed.

**Results:**

In the interviews, 55% of pharmacists acknowledged TCIM as a supportive measure for modern medicine, and 45% responded that TCIM was efficacious. However, pharmacists’ evaluation levels of Kampo medicine were high, whereas pharmacists’ attitudes towards dietary supplements were primarily negative. There have been various proposals to improve the eJIM, such as highlighting important information and providing more specific information on TCIM in Japan. An internet survey of 365 pharmacists showed that 67.4% were consulted by patients regarding TCIM. Of these TCIM, pharmacists’ evaluation levels of Kampo medicines were high. Only 5% of the respondents had visited the eJIM website prior to the survey, and the overall usability score of each web page was high.

**Conclusions:**

Kampo medicines and dietary supplements are common TCIMs that pharmacists use or advise on in Japan. Pharmacists’ evaluation levels of Kampo medicine were high, whereas pharmacists’ attitudes towards dietary supplements were generally negative.

## Background

Traditional, complementary, and integrative medicine (TCIM) has become increasingly important in modern medicine in terms of facilitating holistic patient care [1]. TCIM includes various products, techniques, clinical practices to prevent or heal illness and promote wellbeing [2]. The Ministry of Health, Labor and Welfare (MHLW) of Japan defines integrative medicine (IM) as follows [3]: IM is a medical treatment conducted under a physician’s supervision, based primarily on modern Western medicine, which aims to further improve the quality of life by combining complementary and alternative medicine with conventional medicine in various fields. The National Center for Complementary and Integrative Health (NCCIH) based in the United States supports the research and development of complementary health practices; however, there is no such counterpart in Japan. Instead, the information site for evidence-based Japanese Integrative Medicine (eJIM) (https://www.ejim.ncgg.go.jp/en/index.html) was launched with the support of the MHLW in March 2014. The main purpose of the eJIM website is to provide reliable information about IM to the general population and healthcare professionals, based on scientific and clinical evidence. The eJIM provides information regarding various TCIM therapies, methods for home remedy ingredient production, effects of health food and dietary supplements, yoga, physical manipulation procedures, and other therapies. The eJIM website can be accessed without an ID or password, can be read in both Japanese and English, and has links to global networks.

Information on Kampo medicines and dietary supplements as representative TCIM in Japan is presented in the eJIM website. The Japanese national health insurance system covers the clinical use of 148 Kampo medicines, which are prescribed by physicians at hospitals and clinics, as is done with Western medicines [4]. The model core curriculum for pharmaceutical education in Japan includes health foods/dietary supplements as well as Kampo medicines and crud drugs, whereas the model core curriculum for medical education includes only Kampo medicines [5]. In addition, health foods or dietary supplements (https://ods.od.nih.gov/factsheets/DietarySupplements-Consumer/) are sometimes prescribed in private clinics, but these are not covered by health insurance [6].

We have previously reported the results of surveys conducted in the general population [7] and in a population of physicians [8] designed to obtain feedback on the usability and value of the eJIM resources. These studies revealed that most people and many physicians were not aware of eJIM. Pharmacists act as intermediaries between physicians and patients; therefore, we believe it would be of great importance to understand the perspectives of pharmacists regarding eJIM and TCIM as well. We surveyed the general population and physicians on the usability of eJIM, which has been improved upon solving the problems and deficiencies. Research on the kinds of perspectives Japanese pharmacists have, will contribute to further improvement of the eJIM. This survey also includes how pharmacists collect information on TCIM and how they feel about the usability of eJIM. Thus, more precise information on TCIM will be provided, leading to the safe and effective use of TCIM. Previous reports [8–11] show physicians having a generally negative attitude regarding patients’ use of TCIM. However, it is unknown whether pharmacists share such opinion. This study aims to elucidate the perspectives of pharmacists on TCIM and the usability of eJIM using an internet-based survey.

## Methods

We conducted a two-stage, mixed-method study [12] comprising semi-structured interviews and an internet-based survey, aimed at pharmacists.

### Interviews

From November 1, 2018, to February 28, 2019, we interviewed 20 pharmacists (average age: 40.0 years; 8 males and 12 females). For purposive sampling, we selected these pharmacists considering their workplaces (hospitals, dispensing pharmacies, or retail pharmacies).

Pharmacists in the Kanazawa area were recruited from the Kanazawa Medical University Hospital, and those in Tokyo area were invited to participate by Ichiro Arai (IA). Interviews were conducted face-to-face. The duration of the interviews was approximately 1 hour. We recorded the pharmacists’ opinions after showing them various pages on the eJIM website. With full knowledge of the attributes and conditions of TCIM, the interviewees were allowed to talk freely about their thoughts on the usability and understandability of the eJIM website and could mention any potential improvements that would benefit these aspects of the resource. Utility and usability were scored 0–10, with 0 being not useful and 10 being very useful.

Interview results were analyzed using verbatim records and field notes. To improve the validity of the analysis, interviewees checked the records, and investigators performed a peer-reviewed examination of the analyses. We performed the analysis of the qualitative interview results (iterative or inductive coding, descriptive, and thematic analysis). According to the method developed by Lofland and Lofland [13], the interview data were analyzed by coding and categorizing the content, after the repeated readings of the transcripts and the field notes to understand the overall content. The validation of the analyzed data was improved by a member confirming with the survey participants and a peer examination with research collaborators. The purpose, privacy protection policy, data handling policy, and freedom of participation were explained orally as well as in writing, and informed consent was obtained. The internet survey questionnaires were developed based on these analyses (mixed methods). The interview analysis was not intended for showing the data quantitatively, but its main purpose was as an exploratory study to develop questionnaires for the following quantitative internet survey. Some interviews were done after the commencement of the internet survey.

### Internet-based survey

The questionnaires for the internet-based survey were designed based on the information gathered during the interview process. We outsourced the questionnaire development to INTAGE Healthcare Inc. (Tokyo, Japan), which has rich experience in designing and conducting surveys aimed at healthcare professionals.

The request e-mail was sent to 2023 pharmacists from the INTAGE Healthcare Inc. database and mailing list. A unique URL was issued to each pharmacist for access to the survey. The survey was conducted nationwide from February 8 to February 18, 2019. The survey consisted of 62 questions, including topics derived from the interviews. The contents of the questionnaire examined the perspectives of pharmacists on the use of TCIM in their daily practice and their impressions of the eJIM website. No personal data were collected.

### Ethical considerations

The interviews and the internet surveys were approved by the institutional review boards of Kanazawa Medical University (No. I342) and Nihon Pharmaceutical University (No. 30–06), respectively.

### Reporting checklists

To guide our study and article preparation, we selected the Consolidated Criteria for Reporting Qualitative Research (COREQ) [14] and the Strengthening the Reporting of Observational Studies in Epidemiology (STROBE) from among the reporting guidelines of the EQUATOR Network (https://www.equator-network.org/reporting-guidelines/).

### Statistical analysis

We calculated the sample size by considering the sex, age, and workplace (hospitals, dispensing pharmacies, or retail pharmacies) of potential respondents. The sample size was calculated based on feasible number of pharmacists among monitor members. This survey was intended to compare the three types of working place (hospital, dispensing pharmacy, and retail pharmacy); the number of pharmacists at retail pharmacy was the least, while the feasible number of collections was approximately 100. Therefore, an n-number of 110 for each group, of a total of 330, was determined. Data were expressed as average and standard deviation (SD). Significant differences were analyzed using a two-sided Student’s t-test and chi-square test with the Microsoft Excel software program.

## Results

### Interviews

The interviewees comprised 20 pharmacists. The mean and standard deviation (mean ± SD) of the pharmacists’ ages was 40.0 ± 10.8 years. There were eight men and 12 women. 18 of the 20 (80%) pharmacists offered (or advised on) TCIM therapies in their daily practices. These TCIM therapies included prescribed Kampo medicines, dietary supplements (DS), and over-the-counter (OTC) Kampo medicines (Table 1). 15 of 18 (83.3%) pharmacists provided or advised on Kampo medicines that were initially prescribed by physicians.

**Table 1:** Attributes of participants in the interview (*n* = 20)

	n	%
Gender		
Male	8	40.0
Female	12	60.0
Gender & Age		
Male 20s and 30s	5	25.0
Male 40s	2	10.0
Male 50s and over	1	5.0
Female 20s and 30s	5	25.0
Female 40s	3	15.0
Female 50s and over	4	20.0
Working place		
Hospital pharmacy	9	45.0
Dispensing pharmacy	4	20.0
Retail pharmacy	7	35.0
Hospital pharmacy (*n =* 9)^a^		
Number of beds	0	0
500 and more	9	100.0
Insurance coverage		
Insurance coverage only	9	100.0
Self funded/Self funded dominated by insurance coverage	0	0
Provision of TCIM		
Yes	18	90.0
No	2	10.0

^a^All of the hospital pharmacists belonged to university hospitals, which had more than 500 beds and handled Kampo medicines covered by insurance only

The interviewed pharmacists’ remarks regarding eJIM were categorized according to language-use, readability, information presentation, and information dissemination. For example, as quoted, “It would be more understandable if important parts are highlighted.”; “More information on Kampo is desirable.”; “Resources for explanation to patients are needed.”; “More information on Japanese situation should be enriched.”; and “Visual information such as illustrations and videos would be helpful.” The solutions proposed by the interviewees were as follows: “Instead of describing information from other institutions, this website should have its own standards and definitions, which will be easier to understand.” and “Kampo medicines should be mentioned at the beginning of the website.”

### Internet-based survey findings

Valid responses were obtained from 365 pharmacists among the initially targeted 2023 pharmacists (response rate: 18.0%).

#### Attributes of the respondents

The respondents comprised 137 men (37.6%) and 228 women (62.4%). The majority of respondents were women in their 20s and 30s (24.1%). The distribution of respondents’ working places were well-balanced among hospital pharmacies, dispensing pharmacies (outside the hospital), and retail pharmacies. In the case of hospital pharmacies, the type of health insurance coverage was only 54.5% (68/125). TCIM was provided in 61.9% (226/365) of the patients (Table 2).

**Table 2:** Attributes of respondents in the internet survey (*n* = 365)

	n	%
Gender		
Male	137	37.6
Female	228	62.4
Gender & Age		
Male 20s & 30s	32	8.8
Male 40s	46	12.6
Male 50s and over	59	16.2
Female 20s & 30s	88	24.1
Female 40s	76	20.8
Female 50s and over	64	17.5
Working place^a^		
Hospital pharmacy	125	34.2
Dispensing pharmacy	139	38.1
Retail pharmacy	101	27.7
Number of beds in case of hospital pharmacy (*n* = 125)		
0	18	14.4
1–19	7	5.6
20–99	20	16.0
100–299	37	29.6
300–499	23	18.4
500 and over	20	16.0
Insurance coverage in case of hospital pharmacy (*n =* 125)		
Insurance coverage only	68	54.4
Self funded	1	0.8
Insurance coverage dominated by self funded	56	44.8
Provision of TCIM		
Yes	226	61.9
No	139	38.1

^a^Working places were balanced among 3 types of pharmacy

#### TCIM information resources utilized by respondents

To access information on TCIM therapies, 55.6% (*n* = 109/196) of the pharmacists attended seminars or lectures, 50.8% (*n* = 98/196) obtained information from medical representatives of pharmaceutical companies, and 36.7% (*n* = 72/196) obtained information from catalogs and journals.

#### Information provided by pharmacists for patients

In the case of TCIM, pharmacists provided information to patients in terms of efficacy (90.3%, *n* = 177/196), safety (66.3%, *n* = 130/196), and precautions for use (45.4%, *n* = 89/196). Respondents expressed the need for more evidence-based information on the efficacy, mechanisms of action, safety, and educational materials that they could use to advise patients.

#### Health hazards for patients as reported by respondents

During the reporting period, 7.1% (*n* = 26/365) of patients using TCIM delayed visiting a doctor based on their own decision, and 2.3% (n = 8/365) did not consult a doctor for adverse events possibly caused by TCIM-use.

#### Pharmacists’ perspectives on TCIM

Pharmacists’ perspectives on Kampo medicines and dietary supplements are summarized in Table 3 and Table 4, respectively. Since Kampo medicines have evidence of efficacy and safety, the frequencies of pharmacists’ evaluations for patients using Kampo medicines were high (40.8%, as efficacious as Western medicine, Table 3). On the other hand, pharmacists’ perspectives on dietary supplements were low (3.8%, as efficacious as Western medicine, Table 4), but they still thought that dietary supplements could ameliorate pain (44.7%, Table 4). There was a significant difference between Kampo medicines and dietary supplements in the rates of positive evaluation of efficacy (40.8% vs. 3.8%, *P* = 0.000). There were no significant differences among the three types of working place.

**Table 3:** Perspectives of pharmacists on Kampo medicines (prescribed, OTC, crude drugs) as per the internet survey^a^

Working place	n	As efficacious as Western medicine	Efficacious as complementary measures	Not directly efficacious, but ameliorate pains	No benefits	Hazardous for Western medicine	Others
total	365	40.8%	40.5%	13.2%	1.9%	1.4%	2.2%
Hospital pharmacy	125	32.0%	45.6%	17.6%	1.6%	2.4%	0.8%
Dispensing pharmacy	139	47.5%	37.4%	11.5%	0.0%	1.4%	2.2%
Retail pharmacy	101	42.6%	38.6%	9.9%	5.0%	0.0%	4.0%

^a^Kampo medicines includes Kampo products prescribed by physicians, over-the-counter (OTC) Kampo products not covered by health insurance, and crude drugs some of which are covered by health insurance

**Table 4:** Perspectives of pharmacists on dietary supplements as per the internet survey^a^

Working place	n	As efficacious as Western medicine	Efficacious as complementary measures	Not directly efficacious, but ameliorate pains	No benefits	Hazardous for Western medicine	Others
total	365	3.8%	32.1%	44.7%	13.7%	2.7%	3.0%
Hospital pharmacy	125	4.8%	31.2%	42.4%	13.6%	4.8%	3.2%
Dispensing pharmacy	139	2.2%	28.1%	49.6%	16.5%	1.4%	2.2%
Retail pharmacy	101	5.0%	38.6%	40.6%	9.9%	2.0%	4.0%

^a^Dietary supplements include amino acids, enzymes, herbs, minerals, vitamins, and many other ingredients

#### Evaluation of the contents of eJIM

Before answering the questionnaire, 51.0% (*n* = 186/365) of respondents had heard of TCIM but did not know the meaning of TCIM, 35.9% (*n* = 131/365) of respondents had no understanding or prior knowledge of TCIM, and 13.2% (*n* = 48/365) were familiar with TCIM. The average score regarding the usability and understandability of the eJIM website as it relates to TCIM practices was 5.0. The average scores of the evaluation of the contents are summarized in Table 5. There were significant differences in the average scores for explanations and webpages between hospital and dispensing pharmacies.

**Table 5:** Evaluation of eJIM website by pharmacists working at different types of pharmacy^a, b^

	Hospital pharmacy (*n =* 125)		Dispensing pharmacy (*n* = 139)		Retail pharmacy (*n* = 101)	
	mean	SD	mean	SD	mean	SD
General	6.1	1.80	5.8	1.67	6.0	1.89
Understanding of TCIM	6.5	1.82	6.3	1.65	6.5	1.83
Consulted about TCIM products/services	6.1	1.81	5.9	1.64	6.1	1.70
Recommendation of TCIM products/services	5.8	1.81	5.6	1.63	5.8	1.55
Understandable explanations	6.0^*^	1.70	5.6	1.50	6.0	1.51
Sufficient explanations	5.9^*^	1.74	5.5	1.48	5.9^*^	1.66
Readable pages	6.0^*^	1.85	5.5	1.47	5.9	1.63
Agreeable contents	5.8	1.75	5.6	1.50	6.0	1.60

^a^Evaluation of the eJIM contents and web pages was expressed as numbers such as 0 (not useful), 1, 2 … and 10 (very useful)

^b^*Significant difference compared to dispensing pharmacy (*P* < 0.05)

#### Communication with patients

A total of 41.1% (*n* = 39/95) of respondents reported consulting with patients regarding the use of TCIM while using other drugs. Of this proportion of respondents, 36.8% (*n* = 35/95) did not recognize the potential added value of TCIM unless consulted by patients in this regard.

When pharmacists were asked whether they were aware of patients’ use of TCIM in clinical practice (respondents: *n* = 95), 36.8% (n = 35/95) responded with a negative answer and only knew when the patient consulted them, 41.1% (*n =* 39/95) provided advice on the use of TCIM when there were concerns about contraindications with other drugs or side effects, and 17.9% (*n* = 17/95) did not seek further information about the TCIM approaches that patients were using. Only 4.2% (*n* = 4/95) were aware of the patients received all TCIM therapies.

We asked the pharmacists whether they were asked about the use of TCIM therapies by patients being treated for cancer. While 15.6% (*n =* 17/122) of the pharmacists provided an affirmative response, 58.2% (*n* = 71/122) answered that the eJIM website was a useful resource (score ≥ 6) during consultation with these cancer patients.

#### Access to eJIM

Only 4.7% (*n =* 17/365) of respondents had accessed the eJIM website prior to participating the survey (hospital pharmacy: 6.4%, *n* = 8/125; dispensing pharmacy: 1.4%, *n* = 2/139; retail pharmacy: 6.9%, *n =* 7/101) .

#### Feedback (to the opinions in interviews)

The following opinions proposed in the preliminary interview survey were supported by respondents: better explanation materials for patients (61.1%, *n* = 223/365), more visual information needed, such as illustrations or videos (59.7%, *n* = 218/365), better highlighting of important points (57.0%, *n* = 208/365), more information needed on Kampo medicines (54.0%, *n* = 197/365), and more specific information needed on Japanese TCIM practices (53.2%, *n* = 194/365).

## Discussion

In this study, we sought to clarify Japanese pharmacists’ perspectives on TCIM. Reports on pharmacists using TCIM in their practices and the general perceptions regarding the use and recommendations of TCIM in different countries [15–26] have shown that pharmacists play important roles in the safe use of TCIM by patients. To the best of our knowledge, there has been no previous internet-based survey conducted relating to TCIM and pharmacists’ perspectives thereof. In summary, Kampo medicines and dietary supplements are common TCIMs that pharmacists use or advise on in Japan. Pharmacists’ evaluation levels of Kampo medicine use were high, whereas pharmacists’ attitudes towards dietary supplements were primarily negative. Approximately 50% of the pharmacists were not aware of their patients’ use of TCIM. Approximately 5% of respondents had accessed the eJIM website prior to their participation in the survey.

A 3-year series of surveys regarding eJIM, including the one used in the present study, found that pharmacists (particularly hospital pharmacists) were more interested in browsing eJIM resources than other survey subjects (e.g., general population, physicians, and others). Herein, we discuss the implications of these findings.

Pharmacists are frequently asked by patients for information regarding medicine-types, and their job is to provide an explanation in an easy-to-understand manner. It seems logical that they would work to develop the ability to think from the patient’s point of view. We are searching for insights into how accurately certain information is conveyed to patients. During the interview process in this study, subject pharmacists stated that ample amounts of relevant insights and discussions, were outlined in eJIM resources. In other words, eJIM may be a particularly informative site in the areas of interest relevant to pharmacists. It is expected that this will be expanded upon (e.g., by translating foreign information into Japanese while incorporating information originating in Japan) and will lead to a widely accepted tool aimed at helping pharmacists in their daily practices. Furthermore, TCIM information would be accurately communicated to the general population through pharmacists.

Three types of pharmacists were surveyed, among whom hospital-based pharmacists were the most interested in browsing the eJIM. Their primary interest was on specific Kampo medicines. In hospitals, for example, pharmacists are often consulted by physicians regarding measures to reduce the side effects of anticancer drugs. Useful information on this subject is currently available in the eJIM resources. When physicians consult pharmacists who specialize in anticancer drugs, eJIM can be a useful tool for such consultations in hospital settings. Pharmacists can convey eJIM information to doctors that will help patients continue difficult treatment regimens, increasing the likelihood of improving patient outcome. Although the average scores (understandable explanations, sufficient explanations, and readable pages in Table 5) by pharmacists of hospital pharmacy were significantly higher than those by pharmacists of dispensing pharmacy, the reason is unclear.

Pharmacists in dispensing pharmacies are often consulted by patients and receive information from patients regarding their experiences of using health foods and dietary supplements while taking prescription drugs. These pharmacists showed greater interest in information related to these aspects of TCIM practices. Historically, OTC Kampo medicines and dietary supplements were sold at dispensing pharmacies before Kampo medicines were approved for ethical use (i.e., prescribed by physicians and covered by national health insurance). Kampo formulation for prescription is equal to ethical Kampo formulation (https://www.nikkankyo.org/seihin/yougo/explanation01.htm#117). Pharmacists in retail pharmacy were more interested in health foods and supplements stocked in retail pharmacies (information on these can also be found in eJIM). Therefore, the requirement for TCIM-related information differs according to a pharmacist’s place of work, and eJIM may be used by pharmacists to help patients safely utilize TCIM and contribute to their treatment by enriching patients’ information and understanding.

Several surveys participated by pharmaceutical students regarding their perspectives on TCIM [27, 28] reported that more than half of the students showed positive feelings toward TCIM, although it depended on the country or cultural situation. In Japan, pharmaceutical students are educated regarding Kampo medicines and dietary supplements, especially since 2002, when the inclusion of Kampo medicine in the core curriculum of pharmaceutical education first began. Some examples of the concepts and methods of Kampo Medicine include “qi-blood-water” systems, tong, and abdominal diagnoses. Therefore, pharmaceutical students are familiar with TCIM practices. Considering this, pharmacists younger than 40 years of age are educated regarding TCIM based on the core curriculum now implemented.

The limitations of the present study were as follows: the number of interviewees and the number of respondents to the internet-based survey was small. For validation of the quantitative findings, the relatively small sample numbers were counterbalanced by the diversity of respondents, such as the differences in workplace situations.

## Conclusions

Almost half of the responding Japanese pharmacists had positive attitudes toward TCIM in general, and their evaluation of Kampo medicines was significantly higher than that of dietary supplements. Most pharmacists did not have prior knowledge about the eJIM website, but they found that it could be useful to obtain information on TCIM after being familiarized with the resources during the survey process. Better communication between pharmacists and patients may be facilitated through the eJIM website. To improve the eJIM website, participating pharmacists suggested that important points should be emphasized on the webpages, and natural Japanese translation is necessary to introduce international information.

## Data Availability

The data that support the findings of this study are available from the corresponding author upon reasonable request.

## Abbreviations

TCIM: Traditional, complementary, and integrative medicine
MHLW: Ministry of Health, Labor and Welfare
eJIM: Evidence-based Japanese Integrative Medicine
IM: Integrative medicine
NCCIH: National Center for Complementary and Integrative Health
DS: Dietary supplements
OTC: Over-the-counter
